# Expression of the putative cannabinoid receptor GPR55 is increased in endometrial carcinoma

**DOI:** 10.1007/s00418-021-02018-4

**Published:** 2021-07-29

**Authors:** Thangesweran Ayakannu, Anthony H. Taylor, Justin C. Konje

**Affiliations:** 1 Faculty of Health and Life Sciences, University of Liverpool, Liverpool, UK; 2 Department of Obstetrics and Gynaecology, Gynaecology Oncology Centre, Liverpool Women’s Hospital, Liverpool Women’s NHS Foundation Trust, Liverpool, UK; 3 Endocannabinoid Research Group, Reproductive Sciences Section, Department of Cancer Studies and Molecular Medicine, University of Leicester, Leicester, UK; 4 Department of Molecular and Cell Biology, University of Leicester, George Davies Centre for Medicine, University Road, Leicester, LE2 7RH Leicestershire UK; 5 Department of Health Sciences, University of Leicester, Leicester, UK

**Keywords:** GPR55, *N*-Acylethanolamine, Cannabinoid receptors, Endometrial cancer, Gene expression, Immunohistochemistry

## Abstract

Although the expression of the putative cannabinoid receptor GPR55 has been shown to be involved in the growth of various tumours and is increased in a number of cancers, its expression has not been examined in patients with endometrial cancer (EC). Quantitative RT-PCR (for mRNA levels) and immunohistochemistry (for protein levels) were used to measure GPR55 expression in patients with Type 1 and Type 2 EC and correlated against cannabinoid receptor (CB1 and CB2) protein levels using non-cancerous endometrium as the control tissue. The data indicated that GPR55 transcript and GPR55 protein levels were significantly (*p* < 0.002 and *p* < 0.0001, respectively) higher in EC tissues than in control tissues. The levels of immunoreactive GPR55 protein were correlated with GPR55 transcript levels, but not with the expression of CB1 receptor protein, and were inversely correlated with CB2 protein expression, which was significantly decreased. It can be concluded that GPR55 expression is elevated in women with EC, and thus could provide a potential novel biomarker and therapeutic target for this disease.

**Supplementary Information:**

The online version contains supplementary material available at 10.1007/s00418-021-02018-4.

## Introduction

The incidence of endometrial cancer (EC) is increasing worldwide, especially in younger women (Lortet-Tieulent et al. 2018). The age-adjusted annual incidence in the USA between 2006 and 2010 was 24.3 per 100,000 women, and in the UK in 2008 it was 19.4 per 100,000 women (Siegel et al. 2013; Wartko et al. 2013). The increased incidence in younger women has been linked to an increased abundance of food and the changes in metabolism (including obesity) associated with increased caloric intake (McDonald and Bender 2019), something that has been known for more than 50 years (Twombly et al. 1961). Although the incidence of EC is increasing in younger women, it remains more prevalent in postmenopausal women, who also have an increased caloric intake. One key player in the control of energy metabolism, is the membrane-derived ligand lysophosphatidylinositol (LPI), which binds avidly to the orphan G protein-coupled receptor GPR55 (Alhouayek et al. 2018). When bound to its receptor, LPI increases insulin secretion by pancreatic islets (Metz 1988) and increases fat deposition in adipose tissue, especially in women (Moreno-Navarrete et al. 2012). Although LPI and its 2-arachidonyl derivative (2-arachidonyl-lysophosphatidylinositol; 2-ALPI) are considered the endogenous ligands for GPR55, other ligands, especially those of the endocannabinoid system (ECS), also bind to or activate this receptor (Ryberg et al. 2007).

Components of the ECS, such as the ligands *N*-arachidonoylethanolamine (anandamide [AEA]) (Devane et al. 1992), 2-arachidonoylglycerol (2-AG), *N*-oleoylethanolamide (OEA) and *N*-palmitoylethanolamide (PEA), have all been reported to be raised in endometrial tissues of women with EC (Guida et al. 2010; Ayakannu et al. 2019b). Furthermore, the main receptors for these ligands, cannabinoid receptor 1 [CB1; cloned in 1988 (Devane et al. 1988)] and cannabinoid receptor 2 [CB2; cloned in 1993 (Munro et al. 1993)] have also been detected in endometrial tissue and their levels shown to be reduced in both Type 1 and Type 2 EC (Ayakannu et al. 2018, 2019b). Endocannabinoid ligand signalling by these two ‘classical’ cannabinoid receptors are known to trigger different pathways in the pathogenesis of some cancers, such as those of the liver and breast (Pisanti et al. 2013). Additionally, cancers such as those of the bladder, pancreas and small intestine and prostate cancer, keratinocyte and neuroblastoma cell lines (Brown 2007) respond to endocannabinoid-like ligands in a non-CB1/CB2-dependent manner (Ayakannu et al. 2013), indicating other potential cellular targets for endocannabinoids in tumours involving cells of this type (Wilkinson and Williamson 2007).

These observations indirectly led to the prediction of a third cannabinoid receptor isoform, termed CB3 (Ryberg et al. 2007), which has now been reclassified as GPR55/LPI1 (Brown 2007; Kihara et al. 2014). Activation of this receptor triggers intracellular effects of some cannabinoid receptor ligands (Henstridge et al. 2010), but not all (Ryberg et al. 2007; Henstridge et al. 2010). This could be because GPR55 displays only 13.5% amino acid homology with CB1 and 14.4% homology with CB2 (Baker et al. 2006), or because its pharmacology is complex (Alhouayek et al. 2018). Nevertheless, GPR55 protein is expressed in many normal tissues of the body, such as the brain, spleen, bone, gastrointestinal tract, pancreas, adipose tissue (Simcocks et al. 2016; Kramar et al. 2017), and in some parts of the female reproductive tract (Henstridge et al. 2011), including the endometrium (Ryberg et al. 2007). Previous studies have demonstrated that GPR55 protein is present in the normal brain, gastrointestinal tract, spleen and adrenals (Sawzdargo et al. 1999; Ryberg et al. 2007; Oka et al. 2010) and its expression is increased in a number of tumours (Ford et al. 2010; Hu et al. 2011; Pineiro et al. 2011; Leyva-Illades and Demorrow 2013; He et al. 2015; Falasca and Ferro 2016). Furthermore, the LPI-GPR55 axis has been reported to have immunological roles where activation of the pathway prevents intraepithelial lymphocyte migration within the wall of the small intestine and prevents T-cell attachment (Sumida et al. 2017). The axis also induces tumour angiogenesis (Hofmann et al. 2015), bone remodelling (Mosca et al. 2021) and bone homeostasis (Whyte et al. 2009), and cancer metastasis (Ford et al. 2010) through immune modulation.

Previously, all components of the ECS (ligands, enzymes and receptors) have been demonstrated to be present in the endometrium and modulated in both estrogen-dependent (Type 1) and estrogen-independent (Type 2) EC (Risinger et al. 2003; Ayakannu et al. 2018, 2019a, b); the levels of the ligands are higher in plasma (AEA and OEA) and endometria (AEA, PEA and OEA) of women with EC compared to non-cancer controls (Guida et al. 2010; Ayakannu et al. 2019b). Subsequently, we have demonstrated that the expression of both classical cannabinoid receptors (CB1 and CB2) are significantly down-regulated in EC (Ayakannu et al. 2018, 2019b; Electronic Supplementary Material, Fig. 1). These data led to a question: if the concentrations of the ligands increase, but the expression of their cognate receptors decrease, then how do these ligands contribute to EC cell growth? The answer is possibly through a non-classical cannabinoid receptor (i.e. TRPV1 for AEA), as has been demonstrated in cell lines (Fonseca et al. 2018), or through increased expression of GPR55, a binder of all the ligands mentioned above and known to increase in many forms of cancer (Andradas et al. 2011). This is especially important since elevated expression of GPR55 and the production of its two most cogent ligands LPI and 2-ALPI have already been demonstrated to be important in the development of ovarian cancer (Sutphen et al. 2004).

Our aim in the present study was therefore to test the second possibility, by examining the expression and distribution of GPR55 at the transcript (mRNA) and protein level in patients with either Type 1 or Type 2 EC and comparing those expressions to that of a control cohort. Finally, we related this expression to the protein expression of CB1 and CB2 in the same patient cohorts.

## Materials and methods

### Participants

The women who took part in this study were undergoing hysterectomy at the University Hospitals of Leicester (UHL) National Health Service (NHS) Trust for either endometrial carcinoma or benign gynaecological conditions such as uterine prolapse. All provided signed written informed consent. The study was approved and conducted according to the guidelines of the Leicestershire, Northamptonshire and Rutland Research Ethics Committee (reference number 06/Q2501/49). Volunteers who were simultaneously on or had previously been on any form of hormonal treatment (such as hormone replacement therapy or the levonorgestrel intrauterine system) for the 3 months prior to surgery or who were on prescription or recreational drugs were excluded. Women with chronic medical conditions such as diabetes mellitus, hypertension, those requiring long-term medication or those diagnosed with any other type of cancer were also excluded.

### Patient characteristics

The details of the patients and how their tissue samples were used are in shown in Table 1. Although there appeared to be subtle differences in age and body mass index (BMI) values between the cancer patients and controls, these differences for the patients whose samples were used for quantitative real-time polymerase chain reaction (qRT-PCR) (Table 1) or in immunohistochemistry (Table 1) were not statistically different. All volunteers were postmenopausal, and the endometria of the control group were all classified as atrophic through histological examination. Postmenopausal samples were studied because they are the largest age group of women affected by EC and also to prevent any potential confounding effects of the menstrual cycle on GPR55 expression.

**Table 1:** Patients’ ages and BMI for biopsies analysed by qRT-PCR or immunohistochemistry

Tissue type	Designation	Age (years)	BMI (kg/m^2^)
qRT-PCR^a^			
Control	Atrophic (6)	60.67 ± 4.27	26.67 ± 6.50
Type 1 EC	Grade 1 (6)	66.17 ± 16.14	33.50 ± 8.92
	Grade 2 (6)	66.50 ± 10.25	32.00 ± 5.97
	Grade 3 (3)	72.67 ± 12.06	35.33 ± 6.11
Type 2 EC	Serous (3)	59.00 ± 3.46	37.67 ± 2.52
	Carcinosarcoma (3)	50.00 ± 5.00	36.67 ± 6.43
Immunohistochemistry			
Control	Atrophic (6)	60.67 ± 4.27	26.67 ± 6.50
Type 1 EC	Grade 1 (6)	62.50 ± 13.90	33.00 ± 8.76
	Grade 2 (6)	65.17 ± 9.86	34.83 ± 5.56
	Grade 3 (6)	66.83 ± 7.88	31.50 ± 3.08
Type 2 EC	Serous (4)	70.25 ± 10.97	33.00 ± 6.83
	Carcinosarcoma (6)	58.33 ± 7.42	35.50 ± 5.99

*EC* endometrial cancer

^a^Only the biopsies from non-malignant control tissues were used for both qRT-PCR and immunohistochemistry studies; additional material was used in the immunohistochemistry studies. The data are presented as the mean ± SD. The number of samples is indicated in parentheses after the designated tissue types. Samples taken from the different groups were not significantly different to the non-malignant control (atrophic endometrium); one-way ANOVA with Dunnett’s post-test indicated no significant differences in either age or BMI

### Sample collection

After hysterectomy, uteri were transported immediately on ice to the histopathology department where a consultant gynaecological histopathologist dissected out two representative biopsies: one for histological confirmation (typing and grading) of clinical diagnosis and immunohistochemistry (IHC) and the other for the measurement of GPR55 transcript (mRNA) levels. Biopsies were washed with phosphate buffered saline (PBS) to remove excess blood and then either immediately stored in RNA*later*^®^ (Life Technologies, Paisley, UK) at −80 °C for RNA extraction or in 10% formalin for histological studies. Representative sections (4 μm) were cut from tissues embedded in paraffin wax and dried onto silane-coated slides microscope slides. After drying, sections were subjected to haematoxylin and eosin (H & E) staining for histological confirmation of disease, which was performed by the consultant gynaecological histopathologist. The tissues were classified using the International Federation of Gynecology and Obstetrics (FIGO) classification system (Mutch 2009) into endometrioid (estrogen-dependent Type 1) and non-endometrioid (estrogen-independent Type 2) cancer. The Type 1 EC tissues were further classified by grade (1, 2 or 3) and the Type 2 tissues into serous or carcinosarcoma (Creasman 2009). All the cancer patients were at stage 1 of disease.

### RNA extraction, cDNA synthesis and quantitative real-time PCR

RNA extractions and cDNA syntheses of the endometrial tissues biopsies (100 mg) were as we previously described (Ayakannu et al. 2015). The cDNA was stored at −20 °C prior to qRT-PCR. Quantitative real-time PCR experiments were performed using the validated human endogenous control assay TaqMan Array 96-well plates consisting of three reference genes, MRPL19 (Hs00608519_m1), PPIA (Hs99999904_m1) and IPO8 (Hs00183533_m1), previously demonstrated to be the correct endogenous genes for atrophic and EC endometrial samples (Ayakannu et al. 2015). All of these were VIC/TAMARA dye labelled assays purchased from Applied Biosystems (Life Technologies, Paisley, Scotland, UK). The human GPR55 (Hs00995276_m1) primers and probes were also purchased from Applied Biosystems, as FAM/MGB dye-labelled probes. RT-minus and no-template controls (NTC) containing DNAse-free water instead of template mRNA were included in each run. No product was synthesised in the NTC or RT-minus samples, confirming the absence of contamination with exogenous or genomic DNA. Each assay had an amplification efficiency of 100% ± 10% (Life Technologies). All the reactions were performed in triplicate (both biological and technical).

### Identification, localisation and histomorphometric analysis of GPR55 protein expression

Immunolocalisation was performed using antibodies against human GPR55 (1:200; Rabbit Polyclonal Anti-GPR55 Receptor [NB110-55498; concentration 1.0 mg/ml], Novus Biologicals Europe, Cambridge Science Park, UK), according to the manufacturer’s instructions. Briefly, after dewaxing in xylene and rehydration through graded alcohols to water, endogenous peroxidase and catalase activity was quenched in 6% H_2_O_2_, washed in water and non-specific binding sites blocked with a solution of phosphate buffered saline (PBS) containing 1% bovine serum albumin (BSA). Non-specific binding sites were further blocked with a 1:20 dilution of normal goat serum dispersed in the same BSA-PBS solution and with avidin–biotin blocking solutions (Avidin–Biotin Blocking kit, Novocastra, Peterborough, Northamptonshire, UK) as instructed by the manufacturer. After overnight incubation with either GPR55 antibodies or an equivalent amount of non-immune rabbit IgG and several washing steps, bound rabbit IgG was detected with biotinylated goat-anti-rabbit IgG antibodies, amplified with avidin–biotin complexed to horseradish peroxidases (Novocastra) and visualised with 3,3′-diaminobenzidine (DAB). Non-reactive sites were visualised with light counterstaining with Meyer’s Haematoxylin (Sigma, Poole, Dorset, UK). Slides incubated with rabbit IgG diluted to the same concentrations as the primary antibody were used as negative controls and human pancreas was used as a positive control tissue (Henstridge et al. 2016; Tudurí et al. 2017) (Fig. 3). All samples were processed in a single run to avoid any inter-assay variation and repeated twice to assure reproducibility. Additional staining controls using other positive control tissues are shown in Electronic Supplementary Material, Fig. 2.

Histomorphometric analysis (H-score) of GPR55 expression was performed as we previously described (Ayakannu et al. 2018, 2019a). Images were examined on an Axioplan transmission microscope (Carl Zeiss, Welwyn Garden City, Herts, UK) at ×100 (Plan Neofluar ×10 objective, NA 0.30), ×200 (Plan Neofluar ×20 objective, NA 0.50) and ×400 (Plan Neofluar ×40 objective, NA 0.75) magnification and captured on a Sony DXC-151P 2/3 inch CCD camera mapping to 768 × 493 pixels (Sony Corp., Kanagawa-Ken, Japan). Images were acquired and captured in the presence of daylight and medium neutral density filters with the lamp set at 6400 K. The output.zvi files were converted to .tif images and then analysed using image analysis software (ImageScope version 10.2.2.2319; Aperio Technologies, Inc., Vista, CA, USA). The H-score values for the glands and stroma were determined independently and then combined to provide an overall H-score for the entire tissue (Ayakannu et al. 2018).

### Statistical analysis

GraphPad Prism version 6.00 for windows (GraphPad Software, San Diego CA, USA, www.graphpad.com) was used to perform the statistical analyses. Data that were normally distributed were analysed by parametric one-way analysis of variance (ANOVA) with Dunnett’s post-test. Data that were not normally distributed were expressed as medians and inter-quartile ranges (IQR), and comparison between groups performed using either Mann–Whitney *U* test or one-way analysis of variance (ANOVA) followed by the appropriate ad hoc post analysis. When *p* < 0.05 was obtained, the test between variables was considered to be significant. Correlations were performed using Pearson correlation analyses.

## Results

### GPR55 transcript levels

**Fig. 1:**
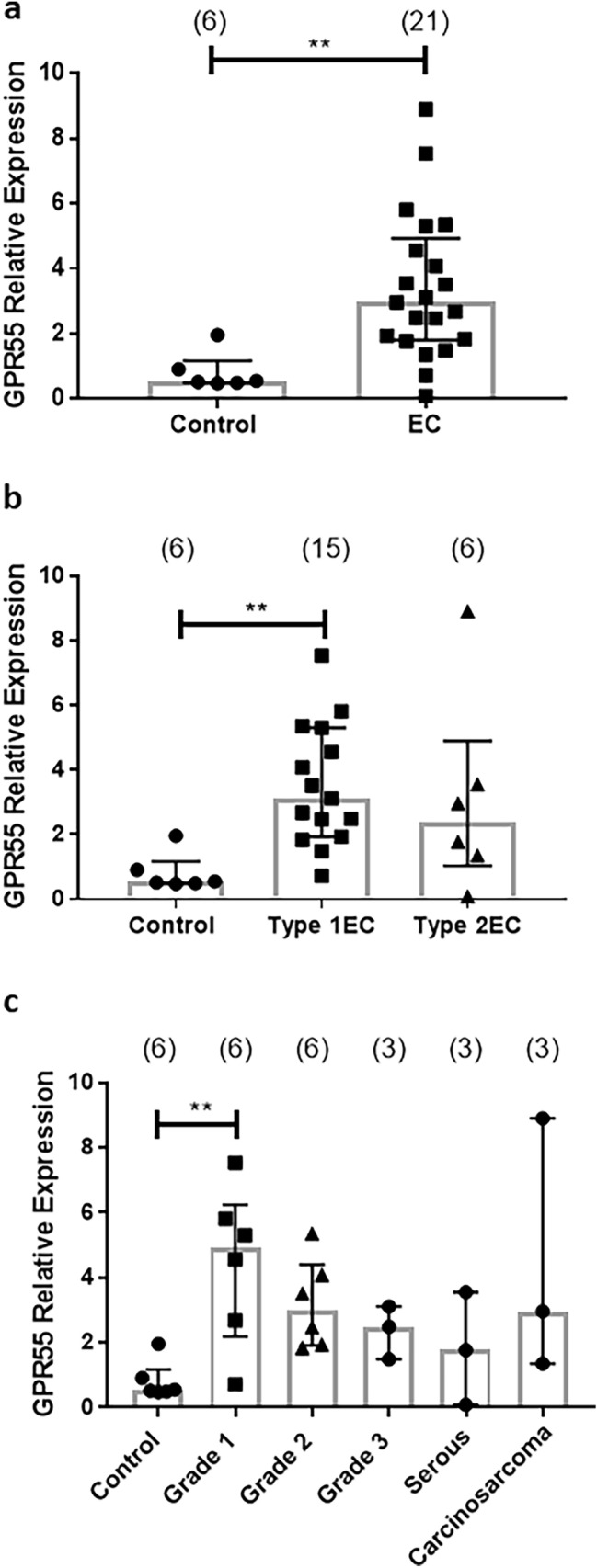
Expression of GPR55 transcript levels in normal and cancerous endometrial tissues. GPR55 transcript levels were normalised against the geometric mean of three ‘housekeeping genes’ and the median of the control endometrium (atrophic) value to provide a relative gene expression value. Data are presented as individual data points for each patient sample together with [median ± (IQR) and range] for control (atrophic), endometrial cancer (EC), Type 1 and Type 2 EC, grades 1, 2 and 3 Type 1 EC tissue, and Type 2 EC (serous and carcinosarcoma) tissues. *p* values were obtained using Mann–Whitney *U* test (**a**; **p = 0.002) and Kruskal–Wallis ANOVA with Dunn’s ad hoc post-test (**b**; **p = 0.007 and **c**; **p = 0.009)

GPR55 transcript (mRNA) levels in EC tissues were three times as high as in the control (atrophic endometria obtained from post-menopausal women without cancer; *n* = 6) (*p* = 0.002) (Fig. 1a). Sub-analysis indicated that GPR55 levels in the estrogen-dependent Type 1 EC tumours (*n* = 15) were responsible for this increased expression (Fig. 1b). Although GPR55 transcript levels were higher in the estrogen-independent Type 2 EC tumours (*n* = 6), this was not statistically significant (Fig. 1b, c). Further analysis of GPR55 transcript levels showed that only the earliest form of Type 1 EC (grade 1) was significantly higher than the levels found in the controls (Fig. 1c).

### Identification and location of GPR55 protein

**Fig. 2:**
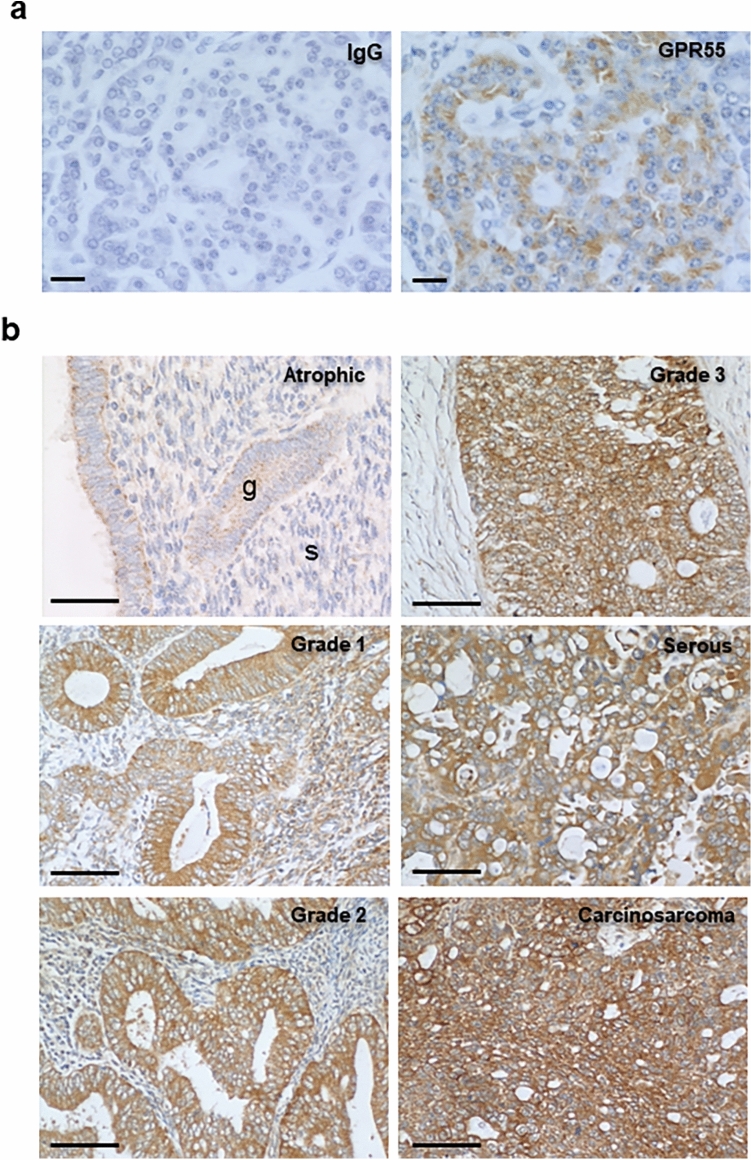
Specificity of GPR55 immunostaining and distribution in normal endometrium and different types of endometrial cancer tissue. Panel **a** shows immunohistochemical specificity for the anti-human GPR55 antibody using human pancreas as the control tissue. Cell-specific staining was observed within the Islets of Langerhans at an antibody dilution of 1 in 200 (GPR55) whilst an equivalent concentration of non-human rabbit IgG showed no staining (IgG). A higher concentration of antibody (1 in 100 dilution) lightly stained acinar cells indicating non-specific staining. Panel **b** shows representative staining of human endometrium from control (atrophic), and Type 1 EC (grades 1, 2 and 3) and Type 2 EC (serous and carcinosarcoma) biopsies. Weak staining in the epithelial glands (g) of control tissue was observed with little or no staining in the stroma (s). By contrast, more intense glandular epithelial cell staining was observed in the different malignant tissues, with some light staining of the stroma in the grade 1, Type 1 EC samples. Bar = 50 µm

The pattern of GPR55 immunoreactivity in representative control (atrophic) and EC tissues and positive control tissue (human pancreas) is shown in Fig. 2. To ensure that the antibody used was specific for GPR55 and the concentration optimal for immunohistochemical (IHC) studies; we performed IHC studies on human pancreas in the absence of a GPR55-specific antibody (IgG; panel a), which showed no DAB staining, whereas in the presence of the GPR55 antibody (GPR55; panel a), β cells within the Islets of Langerhans showed moderate DAB staining. GPR55-specific staining of control endometrial tissue (atrophic; panel b) indicated the presence of very light staining in both the stroma and glands; staining in the luminal epithelial cells was observed on both the apical and basal surfaces. GPR55 protein was present at a stronger intensity in the glands than in the stroma, with the strongest glandular immunoreactivity on the luminal and basal surfaces, with little cytoplasmic staining. By contrast, stromal cell immunoreactivity was very light and not uniform.

GPR55 staining in Type 1 EC tissue was greater than in the control tissue, and was not present in the nucleus but appeared to be localised to the epithelial cell membrane and underlying cytoplasm of both glandular epithelial and stromal cells, where the staining was more intense than in the membranes. Overall stronger staining was observed in the glands than in the stroma. Similar data were obtained for grade 1, grade 2 and grade 3 Type 1 EC samples (panel b). For Type 2 EC (serous and carcinosarcoma), the staining was more uniform and intense than in Type 1 EC samples. GPR55 staining in serous EC samples, was stronger in the glandular epithelial than in the stromal cells, whilst in carcinosarcoma, immunoreactive staining was more uniform and very intense in all cell types (panel b). In both cases, the staining appeared over the entire tissue but was absent from the nuclei of the tumour cells.

### Histomorphometric quantification of GPR55 staining

**Fig. 3:**
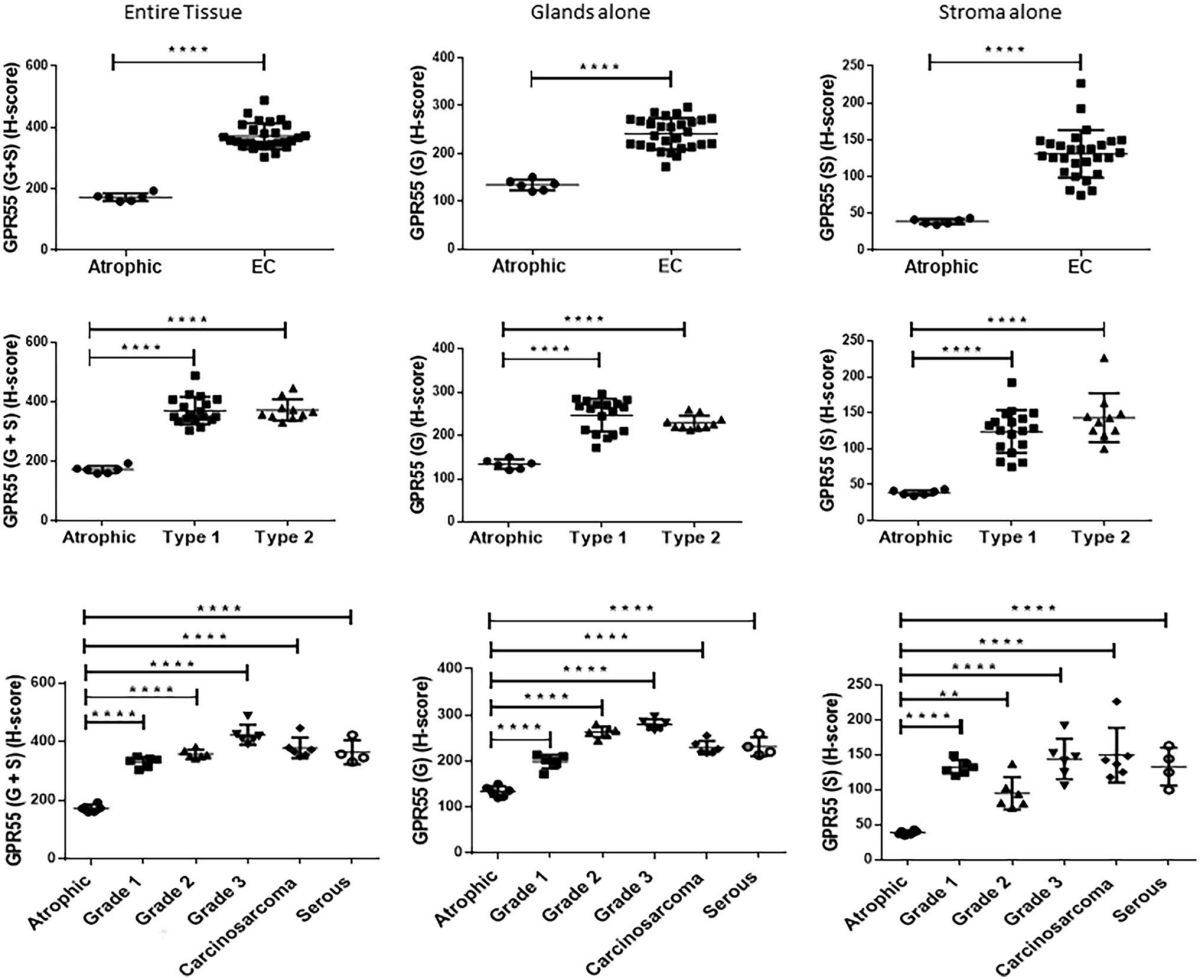
Histomorphometric (H-score) analysis of GPR55 protein immunoreactivity. Immunoreactive GPR55 protein staining (shown in Fig. 2) was subjected to histomorphometric analysis of the entire tissue (left column), the glandular epithelial tissue alone (middle column) and the stromal tissue alone (right column). Data are presented as the mean ± SEM of 6 control (atrophic) and 28 endometrial cancer (EC) tissues. Sub-analyses of endometrial cancer type (Type 1, *n* = 18 and Type 2, *n* = 10) and grade (grade 1, *n* = 6; grade 2, *n* = 6; grade 3, *n* = 6; serous, *n* = 4; carcinosarcoma, *n* = 6) showed that GPR55 immunoreactive protein levels increased both in the stroma and the glandular epithelium in all types and grades of endometrial cancer when compared to that observed in the atrophic control) endometrium. *p* values were obtained using Student’s *t* tests and one-way ANOVA with Dunnett’s ad hoc post-test; **p = 0.0024; ****p < 0.0001

Histomorphometric (H-score) analysis of the GPR55 protein expression (histological staining shown in Fig. 2) in control (atrophic) and EC tissues is shown in Fig. 3. The H-score for GPR55 protein expression was higher in EC samples than in control tissues (upper series of three images). The left-hand panel shows that GPR55 staining was significantly higher (2.1-fold) in the entire tissue [glands (G) and stroma (S)], whilst the middle and right panels indicate that GPR55 staining was significantly increased by 1.8-fold and 3.34-fold in the gland (G) and stromal (S) compartments, respectively. Although the fold changes were different in the two types of endometrial tissue, the staining in the glands (H-score 154.1–248.2) was consistently higher than the staining in the stroma (H-score 39.2–131.0).

Sub-analysis (middle series of three images) of the staining showed that GPR55 protein levels in the Type 1 and Type 2 EC tissues were both significantly increased (2.1-fold) and had similar staining expressions in the glandular epithelial tissue (middle panel) and stromal tissue (right-hand panel). Further analyses of these data indicated that although the H-scores varied between tumour types, GPR55 staining was significantly higher in all grades of Type 1 EC and in both carcinosarcoma and serous EC than in control tissue (lower series of panels).

### GPR55 protein levels correlate with GPR55 transcript levels

**Fig. 4:**
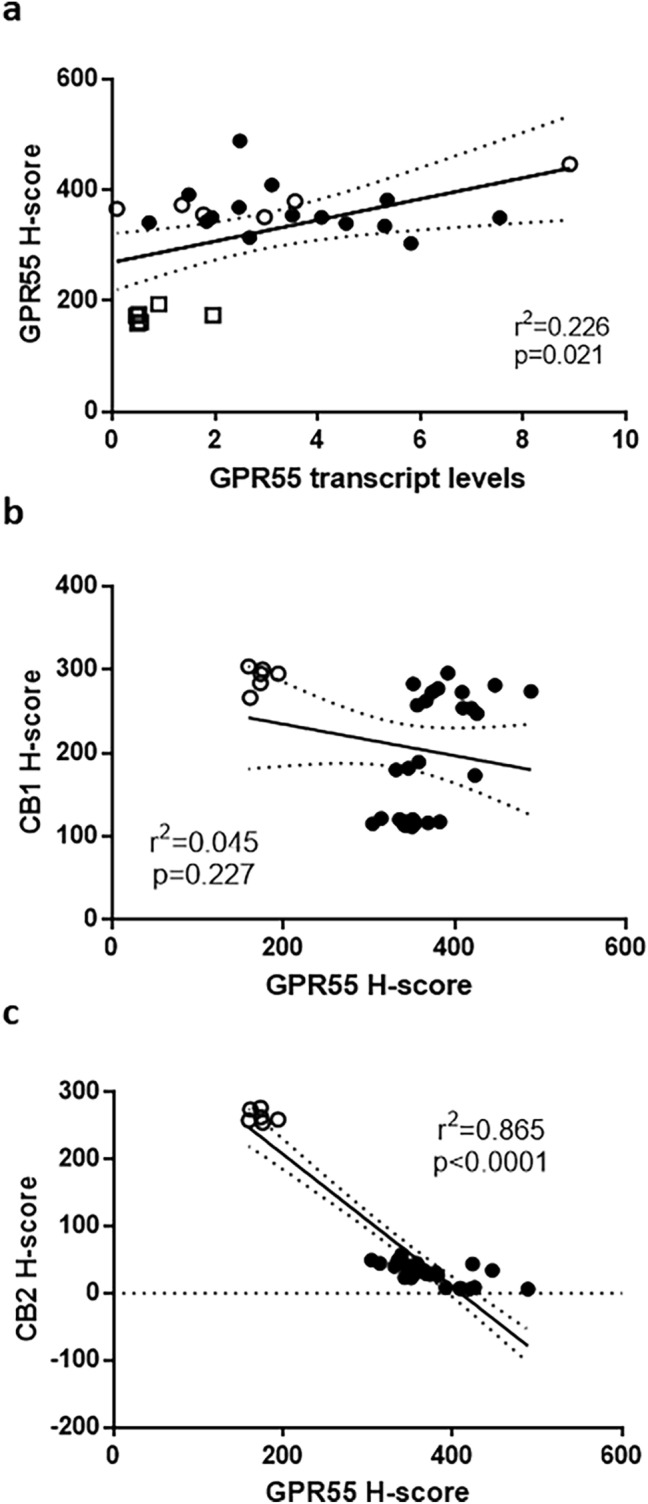
Correlation analysis between GPR55 transcript levels and protein levels and between GPR55 receptor and CB1/CB2 receptor protein levels. Panel **a** shows raw data for the protein levels (H-score) for the entire tissue of control (open squares), Type 1 EC (filled circles) and Type 2 EC (open circles) tissues plotted against transcript levels for the same patient. Panel **b** shows the correlation between GPR55 protein levels (H-score) for the entire tissue of control (atrophic; open circles) or EC (both Type 1 and Type 2 EC; filled circles) plotted against CB1 protein levels (H-score). Panel **c** shows similar data between GPR55 protein levels (H-score) for the entire tissue of control (atrophic; open circles) or EC (both Type 1 and Type 2 EC; filled circles) plotted against CB2 protein levels (H-score) for the same patient. Pearson correlation was performed and the line of best fit (solid line) with the 95% confidence intervals for the regression line (dotted lines) plotted. The correlation coefficient (*r*^2^) and *p *value are also presented. The H-score data for CB1 and CB2 immunohistochemical staining is shown in Electronic Supplementary Material, Fig. 1

Figure 4a shows a clear significant (*p* = 0.02) linear relationship between GPR55 H-scores (protein expression) and GPR55 transcript levels across the entire study. These data suggest that GPR55 protein expression in EC might be regulated primarily at the transcript level.

### Relationship between GPR55 protein expression and cannabinoid receptor protein expression

There was no significant correlation (*r*^2^ = 0.045; *p* = 0.227) between GPR55 protein expression and CB1 protein expression (Fig. 4b), but an almost perfect inverse correlation (*r*^2^ = 0.865; *p* < 0.0001) between GPR55 protein expression and CB2 protein expression (Fig. 4c).

## Discussion

Although several studies have highlighted the role of GPR55 in various types of cancer (Andradas et al. 2011; Pineiro et al. 2011; Perez-Gomez et al. 2013; He et al. 2015; Hofmann et al. 2015), there have been no studies (to our knowledge) on the expression of GPR55 and its potential role in EC. In this study, we mapped out the expression pattern for GPR55 in both Type 1 and Type 2 EC and demonstrated that it was higher in the EC than in control samples and this increased expression was related to tumour type and grade, both at the transcript (*p* = 0.002) and protein (*p* < 0.0001) level. Transcript (mRNA) levels in Type 1 EC patients were significantly higher (*p* < 0.0007), whilst the levels were not affected in Type 2 patients (*p* = 0.132). Further examination of the Type 1 EC patients, revealed that GPR55 transcript levels in grade 1, Type 1 EC tissues were significantly higher than those of grade 2 or 3 patient tissues, whose levels were statistically similar to those of controls, even though they were higher (Fig. 3). These data suggest that different types of EC may have different levels of GPR55 protein and thus differentially respond to GPR55 ligands.

This hypothesis was confirmed by the immunohistochemistry studies, which demonstrated significantly more GPR55 protein in all forms of endometrial cancerous tissue when compared to the control tissues, whilst only the Type 1, grade 1 EC tissue had significantly higher amounts of GPR55 mRNA (Fig. 1). These data indicate that a tight link between GPR55 transcription and translation in malignant endometrial cells is lacking and that there is enhanced protein stability in the Type 1, grade 3 EC, serous and carcinosarcoma samples. This was confirmed with correlation analyses, where the correlation coefficient was only 0.475 (*r*^2^ = 0.226) indicating that GPR55 protein stability is probably greater than GPR55 mRNA stability in EC cells. Staining for GPR55 was not only observed in the plasma membrane where most GPR proteins are normally expressed (Weinberg and Puthenveedu 2019) but also in the cytoplasm where they are not normally expressed. In rapidly dividing tissues, such as ovarian cancer, hepatoma, pancreatic cancer (which are situations where there is increased expression of GPR55) and EC, GPR55 staining might also be observed in the cytoplasm, because during rapid protein synthesis and receptor turnover, GPR55 protein would be observed on ribosomes attached to the rough endoplasmic reticulum, and in vacuoles when the protein is recycled (Weinberg and Puthenveedu 2019).

It was evident that GPR55 protein staining was markedly more intense in the EC tissues, and increased in the more advanced (Type 1, grade 3 and metastatic Type 2) EC tissues (Figs. 2 and 3). There was some evidence of differential expression of GPR55 in the stromal and glandular compartments in the different EC grades and types with stromal expression appearing to be lower in Type 1 EC when compared to Type 2 EC, and the stromal grade 2 expression appearing to be lower than all other types of EC; however, statistical analysis of the difference between Type 1 and Type 2 EC, and between grade 2 and all other types of EC were not significantly different. The key observation from the histomorphometric analyses of the staining patterns was that GPR55 staining was elevated in all forms of EC when compared to non-cancerous tissue (i.e. the atrophic controls). These data suggest that GPR55 expression may play a role in the aetiopathogenesis of EC and may be a possible novel EC biomarker and/or potential future therapeutic target. This suggestion is based on recent evidence, whereby GPR55 has been demonstrated to be an essential/key player in the molecular machinery involved in the regulation and modulation of the signalling pathways responsible for malignant transformation, tumour growth and progression (Dorsam and Gutkind 2007), especially as GPR55 expression correlates in a tumour ‘aggressiveness’-related manner (Andradas et al. 2011). The highest expression of GPR55 protein was observed in samples from the Type 2 EC group (Figs. 2 and 3), which is considered a highly aggressive tumour (Wild et al. 2012). These data suggest that increased GPR55 expression in EC tissue could possibly be considered a marker of EC ‘aggressiveness’, as has been reported for other forms of cancer (Andradas et al. 2011; Hu et al. 2011; Pineiro et al. 2011). Because some endocannabinoids do not bind effectively to GPR55 (Ryberg et al. 2007), the expression of the classical cannabinoid receptors were examined for any evidence of an association with GPR55 expression. There was none with CB1 (Fig. 4b), but a strong inverse correlation with CB2 transcript (data not shown) and protein levels (Fig. 4c). These data suggest that factors regulating GPR55 and CB2 protein expression in EC may be linked in a reciprocal manner. This is important because in EC, the expression of both CB1 and CB2 receptors decrease (Ayakannu et al. 2018) whilst tissue levels of the ligands that bind to and activate these receptors increase (Ayakannu et al. 2019b). Our working hypothesis is that perturbation in the endocannabinoid system in EC results in global gene expression changes that regulate the expression of CB1, CB2 and GPR55 whilst altering the expression of key enzymes that regulate tissue ligand levels (Ayakannu et al. 2019a). Future studies should examine the key regulators of these proteins in relation to EC and other cancers. One potential candidate class of molecules that might regulate both OEA production and GPR55 expression are the lysophospholipids, such as LPI and 2-ALPI (Oka et al. 2007), recently identified as potent ligands for GPR55 (Okuno and Yokomizo 2011). These lipids are noticeably elevated in ascitic fluid obtained from ovarian cancer patients compared to non-malignant controls (Xiao et al. 2001), suggesting a role for GPR55 in ovarian cancer. This lends itself to the possibility that lysophospholipids might be present (or elevated) in the peritoneum of EC patients too. In addition to affecting the tumour cells directly, LPI derived from ovarian cancer cells and ovarian cancer cell lines cause endothelial cell proliferation through binding to GPR55 causing activation of ERK1/2 and p38 (Hofmann et al. 2015). Since ovarian epithelial cancer cells also express GPR55, then a positive feed-forward activation of cell proliferation is postulated. If a similar effect occurs in patients with EC and is confirmed in further studies, then it offers a new opportunity for GPR55 to be considered for the development of anti-angiogenic strategies for patients with both types of gynaecological cancer.

Further support for a feed-forward pathway in carcinogenesis comes from studies of other types of cancer cells where GPR55 is also upregulated. In prostate (PC-3, DU145 and LNCaP) and ovarian (OVCAR3 and A2780) cancer cell lines, an autocrine loop that involves GPR55, LPI and the ABC transporter ABCC1 has been demonstrated (Pineiro et al. 2011), whilst GPR55 expression enhances the invasion and migration of human breast cancer cells (Ford et al. 2010). Conversely, silencing RNA (Perez-Gomez et al. 2013) and microRNA miR-675-5p GPR55 knockdown studies (He et al. 2015), have shown that cancer cell proliferation and colony development in skin carcinoma and non-small lung cancer cells, respectively, is inhibited. Similarly, miR-34p-3p acts as a mediator of p53 modulation of GPR55 expression in pancreatic cancer (Ferro et al. 2018). In those studies, artificial ligands were used, whereas more physiologically relevant ligands might have been more useful. For example, LPI and its 2-arachidonolyl lysophosphatidylinositol (2-ALPI) derivative, are the most potent ligands for GPR55 (Okuno and Yokomizo 2011) with the latter being markedly elevated in ascites fluid from ovarian cancer patients (Xiao et al. 2001; Xu et al. 2001). This has led to the GPR55 receptor being re-labelled the ‘LPI_1_ receptor’ (Kihara et al. 2014). This rebranding could be premature, because the endocannabinoid ligands, 2-AG, PEA and OEA also bind to GPR55 and act as agonists (Ryberg et al. 2007). Where AEA is reported to bind to the GPR55 receptor, it acts as an inverse antagonist, blocking the actions of other endocannabinoids (Ross 2009). Although the *N*-acylethanolamines (NAEs) are eicosanoid lipids and derived from membranes, they are not lysophosphatidylinositol and so not part of this class of ligands. Until such controversies are resolved, we suggest that this receptor continues to be labelled the GPR55 receptor.

Since 2-AG, PEA and AEA are elevated in the plasma of patients with EC and have been demonstrated to induce apoptosis in EC cells (Guida et al. 2010; Fonseca et al. 2018), it seemed prudent to investigate the expression of GPR55 and its relationship with ligands and classical CB1 and CB2 receptors in EC. The clear inverse relationship between the expression of GPR55 and CB2 receptor expression, but not with that of CB1 suggests that the molecular regulation between GPR55 and CB2 may be intricately linked whilst that between GPR55 and CB1 may not be.

Of the endocannabinoids, *N*-palmitoylethanolamide (PEA) has the greatest affinity for this receptor, although other endocannabinoids also bind to GPR55 (Ryberg et al. 2007). It is therefore unsurprising that the correlation coefficients for the interactions between GPR55 protein expression and tissue NAE levels was with PEA > AEA > OEA (data not shown), which suggests that each of these NAEs possesses the potential to regulate the expression GPR55 in EC. This cannot be through CB1 or CB2 because the expression of these receptors decrease to almost zero in EC (Ayakannu et al. 2018, 2019b) and so suggest that activation of another receptor is implicated in EC. Of the various candidates, peroxisome proliferator-activated receptor alpha (PPARα) is the most attractive, since PEA binds to PPARα with high affinity and when activated, PPARα acts as a transcriptional regulator, potentially able to alter the expression of GPR55, CB2 and the enzymes involved in the formation and degradation of the endocannabinoids (Lo Verme et al. 2005). Furthermore, the p53 protein product is known to regulate GPR55 in mice, and since p53 is often mutated in the more aggressive serous and carcinosarcoma forms of EC, then a possible link between mutated p53, the ECS and GPR55 expression is possible. These hypotheses need testing and verifying in future experiments and human studies.

One limitation of this study was that we did not measure the levels of LPI and its congeners in our patient cohorts or other receptors and enzymes that may be important in the GPR55 signalling pathway. This is because it is technically difficult to measure LPI or its congeners in tissues with any degree of accuracy due to rapid degradation of the lipid especially if the fatty acid is unsaturated or if a sample is complicated by the presence of other phospholipids (Xiao et al. 2001; Okuno and Yokomizo 2011; Barr et al. 2012). Furthermore, it is currently known that PPARα and β expression are not altered in EC, but the expression of PPARγ is (Huang et al. 2016). These transcriptional regulators are also involved in glucose metabolism (Wallbillich et al. 2017) and interact with LPI and STAT3, factors that are also known to be a control point in obesity progression (Chang et al. 2015; Arifin and Falasca 2016), which in turn has a strong relationship to the development and progression of EC (Onstad et al. 2016). Another factor that we have not examined is whether the expression of GPR55 has any effect on patient survival or prognosis, but these are matters for future research.

The demonstration that GPR55 expression in EC tissue is enhanced in a way that favours the support of EC cell survival and proliferation and especially in more aggressive tumour types, similar to that observed for ovarian cancer (Sutphen et al. 2004) suggests a possible pivotal role for this receptor in EC pathogenesis. The demonstration that GPR55 expression is increased in both types of EC, especially in the more aggressive Type 2 form, may also provide additional prognostic markers and therapeutic targets (Alhouayek et al. 2018) for individualised treatment and give hope for those women with metastatic disease. As more data become known about how this protein’s expression is regulated, then new windows of opportunity to explore its role as a prognostic marker in response to therapy may become important.

## Supplementary Information

Below is the link to the electronic supplementary material.

Supplementary file1 (DOCX 5531 KB)

Supplementary file2 (DOCX 9278 KB)

## Data Availability

The data sets generated or analysed during this study are included in this published article. Additional information is available from the corresponding author upon reasonable request.

## References

[CR1] Alhouayek M, Masquelier J, Muccioli GG (2018). Lysophosphatidylinositols, from cell membrane constituents to GPR55 ligands. Trends Pharmacol Sci.

[CR2] Andradas C, Caffarel MM, Perez-Gomez E, Salazar M, Lorente M, Velasco G, Guzman M, Sanchez C (2011). The orphan G protein-coupled receptor GPR55 promotes cancer cell proliferation via ERK. Oncogene.

[CR3] Andradas C, Caffarel MM, Pérez-Gómez E, Guzmán M, Sánchez C (2013) The role of GPR55 in cancer. In: Abood M, Sorensen R, Stella N (eds) endoCANNABINOIDS. Springer, New York

[CR4] Arifin SA, Falasca M (2016). Lysophosphatidylinositol signalling and metabolic diseases. Metabolites.

[CR5] Ayakannu T, Taylor AH, Marczylo TH, Willets JM, Konje JC (2013). The endocannabinoid system and sex steroid hormone-dependent cancers. Int J Endocrinol.

[CR6] Ayakannu T, Taylor AH, Willets JM, Brown L, Lambert DG, Mcdonald J, Davies Q, Moss EL, Konje JC (2015). Validation of endogenous control reference genes for normalizing gene expression studies in endometrial carcinoma. Mol Hum Reprod.

[CR7] Ayakannu T, Taylor AH, Konje JC (2018). Cannabinoid receptor expression in estrogen-dependent and estrogen-independent endometrial cancer. J Recept Signal Transduct Res.

[CR8] Ayakannu T, Taylor AH, Bari M, Mastrangelo N, Maccarrone M, Konje JC (2019). Expression and function of the endocannabinoid modulating enzymes fatty acid amide hydrolase and N-acylphosphatidylethanolamine-specific phospholipase D in endometrial carcinoma. Front Oncol.

[CR9] Ayakannu T, Taylor AH, Marczylo TH, Maccarrone M, Konje JC (2019). Identification of novel predictive biomarkers for endometrial malignancies: N-acylethanolamines. Front Oncol.

[CR10] Baker D, Pryce G, Davies WL, Hiley CR (2006). In silico patent searching reveals a new cannabinoid receptor. Trends Pharmacol Sci.

[CR11] Barr J, Caballería J, Martínez-Arranz I, Domínguez-Díez A, Alonso C, Muntané J, Pérez-Cormenzana M, García-Monzón C, Mayo R, Martín-Duce A, Romero-Gómez M, Lo Iacono O, Tordjman J, Andrade RJ, Pérez-Carreras M, Le Marchand-Brustel Y, Tran A, Fernández-Escalante C, Arévalo E, García-Unzueta M, Clement K, Crespo J, Gual P, Gómez-Fleitas M, Martínez-Chantar ML, Castro A, Lu SC, Vázquez-Chantada M, Mato JM (2012). Obesity-dependent metabolic signatures associated with nonalcoholic fatty liver disease progression. J Proteome Res.

[CR12] Brown AJ (2007). Novel cannabinoid receptors. Br J Pharmacol.

[CR13] Chang CC, Wu MJ, Yang JY, Camarillo IG, Chang CJ (2015). Leptin-STAT3-G9a signaling promotes obesity-mediated breast cancer progression. Cancer Res.

[CR14] Creasman W (2009). Revised FIGO staging for carcinoma of the endometrium. Int J Gynaecol Obstet.

[CR15] Devane WA, Dysarz FA, Johnson MR, Melvin LS, Howlett AC (1988). Determination and characterization of a cannabinoid receptor in rat brain. Mol Pharmacol.

[CR16] Devane WA, Hanus L, Breuer A, Pertwee RG, Stevenson LA, Griffin G, Gibson D, Mandelbaum A, Etinger A, Mechoulam R (1992). Isolation and structure of a brain constituent that binds to the cannabinoid receptor. Science.

[CR17] Dorsam RT, Gutkind JS (2007). G-protein-coupled receptors and cancer. Nat Rev Cancer.

[CR18] Falasca M, Ferro R (2016). Role of the lysophosphatidylinositol/GPR55 axis in cancer. Adv Biol Regul.

[CR19] Ferro R, Adamska A, Lattanzio R, Mavrommati I, Edling CE, Arifin SA, Fyffe CA, Sala G, Sacchetto L, Chiorino G, De Laurenzi V, Piantelli M, Sansom OJ, Maffucci T, Falasca M (2018). GPR55 signalling promotes proliferation of pancreatic cancer cells and tumour growth in mice, and its inhibition increases effects of gemcitabine. Oncogene.

[CR20] Fonseca BM, Correia-Da-Silva G, Teixeira NA (2018). Cannabinoid-induced cell death in endometrial cancer cells: involvement of TRPV1 receptors in apoptosis. J Physiol Biochem.

[CR21] Ford LA, Roelofs AJ, Anavi-Goffer S, Mowat L, Simpson DG, Irving AJ, Rogers MJ, Rajnicek AM, Ross RA (2010). A role for L-alpha-lysophosphatidylinositol and GPR55 in the modulation of migration, orientation and polarization of human breast cancer cells. Br J Pharmacol.

[CR22] Guida M, Ligresti A, de Filippis D, Damico A, Petrosino S, Cipriano M, Bifulco G, Simonetti S, Orlando P, Insabato L, Nappi C, di Spiezio Sardo A, di Marzo V, Iuvone T (2010). The levels of the endocannabinoid receptor CB2 and its ligand 2-arachidonoylglycerol are elevated in endometrial carcinoma. Endocrinology.

[CR01] He D, Wang J, Zhang C, Shan B, Deng X, Li B, Zhou Y, Chen W, Hong J, Gao Y, Chen Z, Duan C (2015). Down-regulation of miR-675-5p contributes to tumor progression and development by targeting pro-tumorigenic GPR55 in non-small cell lung cancer. Mol Cancer.

[CR23] Henstridge CM, Balenga NA, Schroder R, Kargl JK, Platzer W, Martini L, Arthur S, Penman J, Whistler JL, Kostenis E, Waldhoer M, Irving AJ (2010). GPR55 ligands promote receptor coupling to multiple signalling pathways. Br J Pharmacol.

[CR24] Henstridge CM, Balenga NA, Kargl J, Andradas C, Brown AJ, Irving A, Sanchez C, Waldhoer M (2011). Minireview: recent developments in the physiology and pathology of the lysophosphatidylinositol-sensitive receptor GPR55. Mol Endocrinol.

[CR25] Henstridge CM, Brown AJ, Waldhoer M (2016). GPR55: metabolic help or hindrance?. Trends Endocrinol Metab.

[CR26] Hofmann NA, Yang J, Trauger SA, Nakayama H, Huang L, Strunk D, Moses MA, Klagsbrun M, Bischoff J, Graier WF (2015). The GPR 55 agonist, L-alpha-lysophosphatidylinositol, mediates ovarian carcinoma cell-induced angiogenesis. Br J Pharmacol.

[CR27] Hu G, Ren G, Shi Y (2011). The putative cannabinoid receptor GPR55 promotes cancer cell proliferation. Oncogene.

[CR28] Huang Q, Zhang H, Chen YJ, Chi YL, Dong S (2016). The inflammation response to DEHP through PPARgamma in endometrial cells. Int J Environ Res Public Health.

[CR29] Kihara Y, Maceyka M, Spiegel S, Chun J (2014). Lysophospholipid receptor nomenclature review: IUPHAR Review 8. Br J Pharmacol.

[CR30] Kramar C, Loureiro M, Renard J, Laviolette SR (2017). Palmitoylethanolamide modulates GPR55 receptor signaling in the ventral hippocampus to regulate mesolimbic dopamine activity, social interaction, and memory processing. Cannabis Cannabinoid Res.

[CR31] Life Technologies. *Amplification efficiency of TaqMan® gene expression assays.* Life Technologies Application Note:lifetechnologies.com. PG1397-PJ6287-CO010377-Amplification Efficiency Assays AppNote-Americas.indd (thermofisher.com). Accessed 13 Apr 2021

[CR32] Leyva-Illades D, Demorrow S (2013). Orphan G protein receptor GPR55 as an emerging target in cancer therapy and management. Cancer Manag Res.

[CR33] Lo Verme J, Fu J, Astarita G, La Rana G, Russo R, Calignano A, Piomelli D (2005). The nuclear receptor peroxisome proliferator-activated receptor-alpha mediates the anti-inflammatory actions of palmitoylethanolamide. Mol Pharmacol.

[CR34] Lortet-Tieulent J, Ferlay J, Bray F, Jemal A (2018). International patterns and trends in endometrial cancer incidence, 1978–2013. J Natl Cancer Inst.

[CR35] McDonald ME, Bender DP (2019). Endometrial cancer: obesity, genetics, and targeted agents. Obstet Gynecol Clin N Am.

[CR36] Metz SA (1988). Mobilization of cellular Ca2+ by lysophospholipids in rat islets of Langerhans. Biochim Biophys Acta.

[CR37] Moreno-Navarrete JM, Catalan V, Whyte L, Diaz-Arteaga A, Vazquez-Martinez R, Rotellar F, Guzman R, Gomez-Ambrosi J, Pulido MR, Russell WR, Imbernon M, Ross RA, Malagon MM, Dieguez C, Fernandez-Real JM, Fruhbeck G, Nogueiras R (2012). The L-alpha-lysophosphatidylinositol/GPR55 system and its potential role in human obesity. Diabetes.

[CR38] Mosca MG, Mangini M, Cioffi S, Barba P, Mariggio S (2021). Peptide targeting of lysophosphatidylinositol-sensing GPR55 for osteoclastogenesis tuning. Cell Commun Signal.

[CR39] Munro S, Thomas KL, Abu-Shaar M (1993). Molecular characterization of a peripheral receptor for cannabinoids. Nature.

[CR40] Mutch DG (2009). The new FIGO staging system for cancers of the vulva, cervix, endometrium and sarcomas. Gynecol Oncol.

[CR41] Oka S, Nakajima K, Yamashita A, Kishimoto S, Sugiura T (2007). Identification of GPR55 as a lysophosphatidylinositol receptor. Biochem Biophys Res Commun.

[CR42] Oka S, Kimura S, Toshida T, Ota R, Yamashita A, Sugiura T (2010). Lysophosphatidylinositol induces rapid phosphorylation of p38 mitogen-activated protein kinase and activating transcription factor 2 in HEK293 cells expressing GPR55 and IM-9 lymphoblastoid cells. J Biochem.

[CR43] Okuno T, Yokomizo T (2011). What is the natural ligand of GPR55?. J Biochem.

[CR44] Onstad MA, Schmandt RE, Lu KH (2016). Addressing the role of obesity in endometrial cancer risk, prevention, and treatment. J Clin Oncol.

[CR45] Perez-Gomez E, Andradas C, Flores JM, Quintanilla M, Paramio JM, Guzman M, Sanchez C (2013). The orphan receptor GPR55 drives skin carcinogenesis and is upregulated in human squamous cell carcinomas. Oncogene.

[CR46] Pineiro R, Maffucci T, Falasca M (2011). The putative cannabinoid receptor GPR55 defines a novel autocrine loop in cancer cell proliferation. Oncogene.

[CR47] Pisanti S, Picardi P, D’alessandro A, Laezza C, Bifulco M (2013). The endocannabinoid signaling system in cancer. Trends Pharmacol Sci.

[CR48] Risinger JI, Maxwell GL, Chandramouli GVR, Jazaeri A, Aprelikova O, Patterson T, Berchuck A, Barrett JC (2003). Microarray analysis reveals distinct gene expression profiles among different histologic types of endometrial cancer. Cancer Res.

[CR49] Ross RA (2009). The enigmatic pharmacology of GPR55. Trends Pharmacol Sci.

[CR50] Ryberg E, Larsson N, Sjogren S, Hjorth S, Hermansson NO, Leonova J, Elebring T, Nilsson K, Drmota T, Greasley PJ (2007). The orphan receptor GPR55 is a novel cannabinoid receptor. Br J Pharmacol.

[CR51] Sawzdargo M, Nguyen T, Lee DK, Lynch KR, Cheng R, Heng HH, George SR, O’dowd BF (1999). Identification and cloning of three novel human G protein-coupled receptor genes GPR52, PsiGPR53 and GPR55: GPR55 is extensively expressed in human brain. Brain Res Mol Brain Res.

[CR52] Siegel R, Naishadham D, Jemal A (2013). Cancer statistics, 2013. CA Cancer J Clin.

[CR53] Simcocks AC, O’keefe L, Hryciw DH, Mathai ML, Hutchinson DS, Mcainch AJ (2016) GPR55. In: Choi S (ed) Encyclopedia of signaling molecules. Springer, New York, pp 1–18

[CR54] Sumida H, Lu E, Chen H, Yang Q, Mackie K, Cyster JG (2017). GPR55 regulates intraepithelial lymphocyte migration dynamics and susceptibility to intestinal damage. Sci Immunol.

[CR55] Sutphen R, Xu Y, Wilbanks GD, Fiorica J, Grendys EC, Lapolla JP, Arango H, Hoffman MS, Martino M, Wakeley K, Griffin D, Blanco RW, Cantor AB, Xiao YJ, Krischer JP (2004). Lysophospholipids are potential biomarkers of ovarian cancer. Cancer Epidemiol Biomark Prev.

[CR56] Tudurí E, Imbernon M, Hernández-Bautista RJ, Tojo M, Fernø J, Diéguez C, Nogueiras R (2017). GPR55: a new promising target for metabolism?. J Mol Endocrinol.

[CR57] Twombly GH, Scheiner S, Levitz M (1961). Endometrial cancer, obesity, and estrogenic excretion in women. Am J Obstet Gynecol.

[CR58] Wallbillich JJ, Josyula S, Saini U, Zingarelli RA, Dorayappan KD, Riley MK, Wanner RA, Cohn DE, Selvendiran K (2017). High glucose-mediated STAT3 in endometrial cancer is inhibited by metformin: Therapeutic implications for endometrial cancer. PLoS One.

[CR59] Wartko P, Sherman ME, Yang HP, Felix AS, Brinton LA, Trabert B (2013). Recent changes in endometrial cancer trends among menopausal-age U.S. women. Cancer Epidemiol.

[CR60] Weinberg ZY, Puthenveedu MA (2019). Regulation of G protein-coupled receptor signaling by plasma membrane organization and endocytosis. Traffic.

[CR61] Whyte LS, Ryberg E, Sims NA, Ridge SA, Mackie K, Greasley PJ, Ross RA, Rogers MJ (2009). The putative cannabinoid receptor GPR55 affects osteoclast function in vitro and bone mass in vivo. Proc Natl Acad Sci U S A.

[CR62] Wild PJ, Ikenberg K, Fuchs TJ, Rechsteiner M, Georgiev S, Fankhauser N, Noske A, Roessle M, Caduff R, Dellas A, Fink D, Moch H, Krek W, Frew IJ (2012). p53 suppresses type II endometrial carcinomas in mice and governs endometrial tumour aggressiveness in humans. EMBO Mol Med.

[CR63] Wilkinson JD, Williamson EM (2007). Cannabinoids inhibit human keratinocyte proliferation through a non-CB1/CB2 mechanism and have a potential therapeutic value in the treatment of psoriasis. J Dermatol Sci.

[CR64] Xiao YJ, Schwartz B, Washington M, Kennedy A, Webster K, Belinson J, Xu Y (2001). Electrospray ionization mass spectrometry analysis of lysophospholipids in human ascitic fluids: comparison of the lysophospholipid contents in malignant vs nonmalignant ascitic fluids. Anal Biochem.

[CR65] Xu Y, Xiao YJ, Baudhuin LM, Schwartz BM (2001). The role and clinical applications of bioactive lysolipids in ovarian cancer. J Soc Gynecol Investig.

