# Supplementary material for: Expression of the putative cannabinoid receptor GPR55 is increased in endometrial carcinoma
Source: Histochem Cell Biol. 2021 Jul 29;156(5):449–60. doi: 10.1007/s00418-021-02018-4 (PMC8604869; doi:10.1007/s00418-021-02018-4)
Supplement: Supplementary file 1 — Supplementary file1 (DOCX 5531 KB) [file 418_2021_2018_MOESM1_ESM.docx]

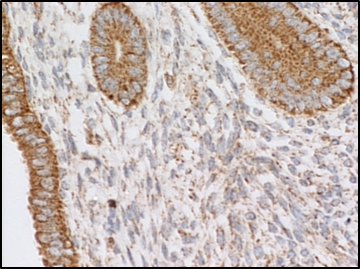

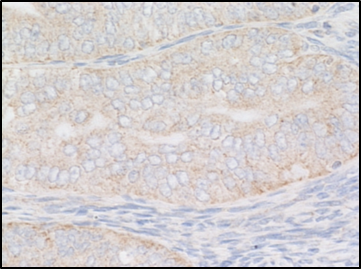

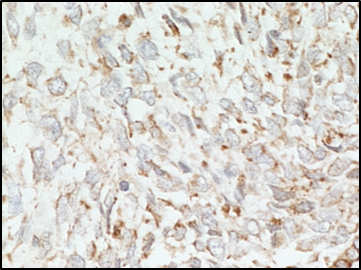

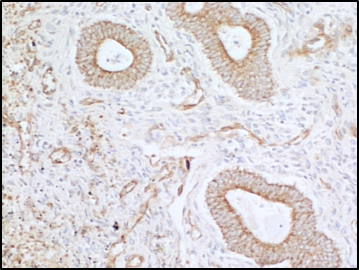

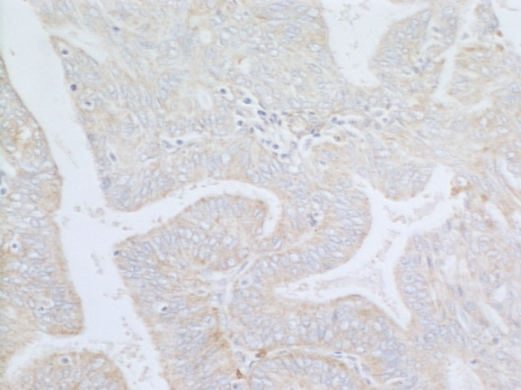

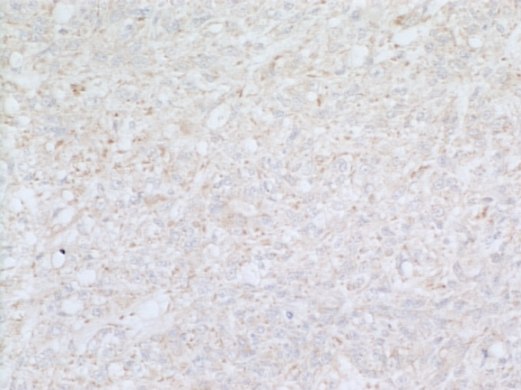


CB2

CB1

Atrophic

control

Type 1

EC

Type 2

EC

**a**

**b**

**Supplemental Figure 1. Immunohistochemical staining patterns and histomorphometric analyses for CB1 receptor and CB2 receptor in normal and EC tissues.**

Panel a shows representative photomicrographs of the staining patterns for the CB1 receptor and the CB2 receptor in normal postmenopausal endometrium (Atrophic control) and malignant tissues taken from women with either Type 1 EC (grade 2) or Type 2 EC (carcinosarcoma). Immunohistochemical staining was performed with commercial antibodies as described (Ayakannu et al. 2018). Briefly, after dewaxing, rehydration and blocking steps, adjacent sections to those used for GPR55 staining were subjected to immunohistochemistry using rabbit anti-human IgG antibodies for CB1 (catalogue number C1108; 1 in 500 dilution) and CB2 (catalogue number C1358; 1 in 150 dilution). Both antibodies were purchased from Sigma Life Science (Poole, Dorset, UK) and used as described (Ayakannu et al. 2018). Image capture and histomorphometric analyses were performed as described in the Materials & Methods section. Bar = 50μm.

Panel b shows the histomorphometric analyses for these samples and the data are presented as the H-score for each individual patient, with the horizontal bar indicating the median and the error bars indicating the IQR, the number of patient samples in each group is shown in parentheses. The data were analysed by one-way ANOVA with Tukey’s HSD test; ****p<0.0001.

The data presented here was used to generate the correlation analyses shown in Figure 4.

**Reference**

Ayakannu T, Taylor AH, Konje JC (2018) Cannabinoid receptor expression in estrogen-dependent and estrogen-independent endometrial cancer. J Recept Signal Transduct Res 38 (5-6):385-392. doi:10.1080/10799893.2018.1531890
