# Supplementary material for: Expression of the putative cannabinoid receptor GPR55 is increased in endometrial carcinoma
Source: Histochem Cell Biol. 2021 Jul 29;156(5):449–60. doi: 10.1007/s00418-021-02018-4 (PMC8604869; doi:10.1007/s00418-021-02018-4)
Supplement: Supplementary file 2 — Supplementary file2 (DOCX 9278 KB) [file 418_2021_2018_MOESM2_ESM.docx]

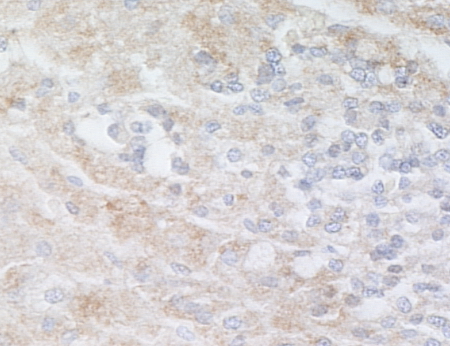

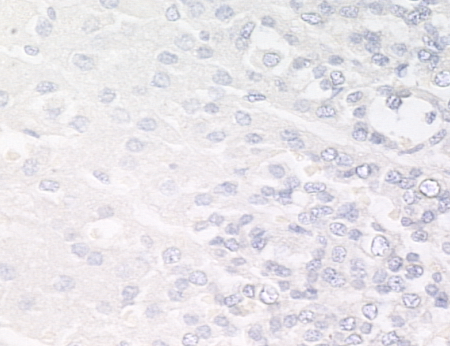


Adrenal


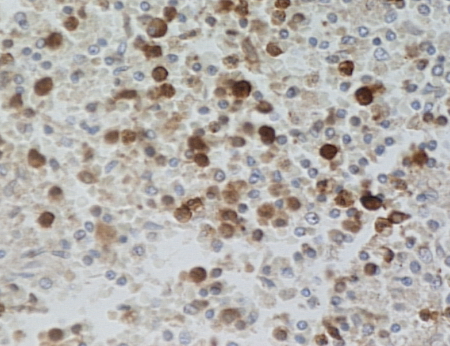


Spleen


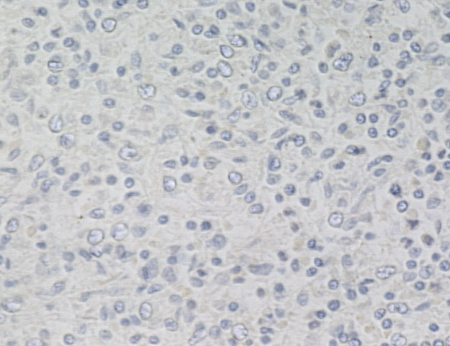

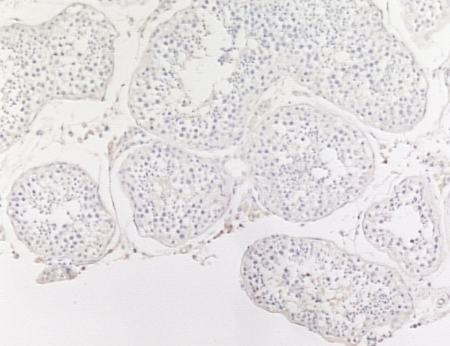

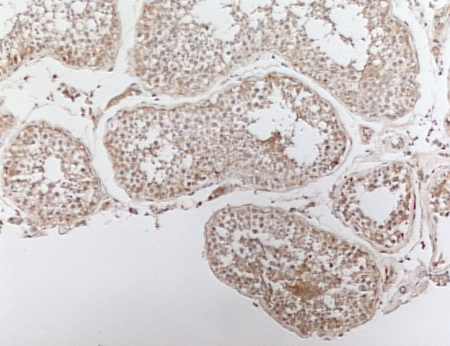


Testis


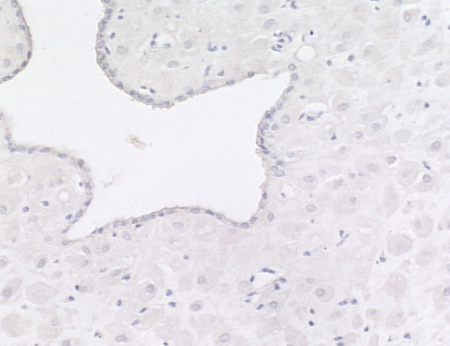

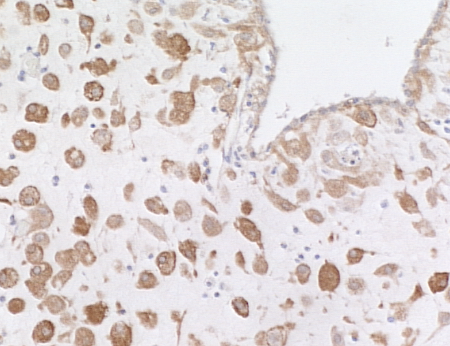


Decidua


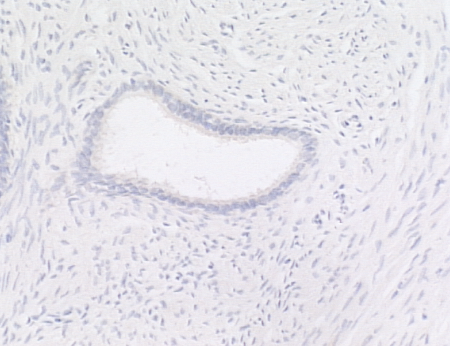

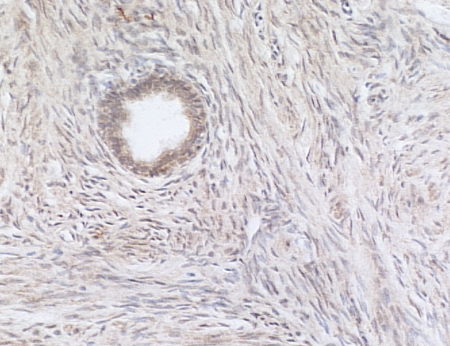


Proliferative

phase

endometrium

Rabbit IgG

GPR55

**Supplemental Figure 2. Immunohistochemical staining for GPR55 in representative positive control tissues**

This figure shows representative photomicrographs of the staining patterns for GPR55 in normal human tissues. Immunohistochemical staining and image capture and analyses were performed as described in the **Materials and Methods** Section. The positive control tissues were chosen based on existing literature (AlSuleimani and Hiley 2015; Fonseca et al. 2011; Henstridge et al. 2016; Ryberg et al. 2007; Shore and Reggio 2015) and by reference to the supplier of the antibody [GPR55 Antibody (NB110-55498): Novus Biologicals](https://www.novusbio.com/products/gpr55-antibody_nb110-55498#reviews-publications) . Bar = 50μm.

**References**

AlSuleimani YM, Hiley CR (2015) The GPR55 agonist lysophosphatidylinositol relaxes rat mesenteric resistance artery and induces Ca(2+) release in rat mesenteric artery endothelial cells. Br J Pharmacol 172 (12):3043-3057. doi:10.1111/bph.13107

Fonseca BM, Teixeira NA, Almada M, Taylor AH, Konje JC, Correia-da-Silva G (2011) Modulation of the novel cannabinoid receptor - GPR55 - during rat fetoplacental development. Placenta 32 (6):462-469. doi:10.1016/j.placenta.2011.03.007

Henstridge CM, Brown AJ, Waldhoer M (2016) GPR55: Metabolic help or hindrance? Trends Endocrinol Metab 27 (9):606-608. doi:10.1016/j.tem.2016.04.012

Ryberg E, Larsson N, Sjogren S, Hjorth S, Hermansson NO, Leonova J, Elebring T, Nilsson K, Drmota T, Greasley PJ (2007) The orphan receptor GPR55 is a novel cannabinoid receptor. Br J Pharmacol 152 (7):1092-1101. doi:10.1038/sj.bjp.0707460

Shore DM, Reggio PH (2015) The therapeutic potential of orphan GPCRs, GPR35 and GPR55. Front Pharmacol 6:69. doi:10.3389/fphar.2015.00069
